# LncRNA MCM3AP-AS1 is downregulated in diabetic retinopathy and promotes cell apoptosis by regulating miR-211/SIRT1

**DOI:** 10.1186/s13098-022-00836-7

**Published:** 2022-05-15

**Authors:** Zhaoxia Xia, Chaoying Yang, Xiaoxi Yang, Shuduan Wu, Zhizhen Feng, Lei Qu, Xianghua Chen, Linyu Liu, Yanling Ma

**Affiliations:** 1 Department of Ophthalmology, the Sixth Affiliated Hospital, Sun Yat-Sen University, No. Two Heng Road 26th, Tianhe District, Guangzhou, Guangdong 510655 People’s Republic of China; 2 Department of Dermatology, the Sixth Affiliated Hospital, Sun Yat-Sen University, Guangzhou, Guangdong 510655 People’s Republic of China

**Keywords:** Diabetic retinopathy, MCM3AP-AS1, miR-211, SIRT1, Apoptosis

## Abstract

**Aim:**

This study aimed to investigate the role of lncRNA MCM3AP-AS1 in diabetic retinopathy (DR).

**Methods:**

Plasma MCM3AP-AS1 levels in DR patients (n = 80), T2DM patients (n = 80), and Controls (n = 80) were measured by qPCR and compared using ANOVA (one-way) and Tukey test. The expressions of lncRNA MCM3AP-AS1 and miR-211 in Human retinal pigment epithelial cells (hRPE) line ARPE-19 were detected by RT-qPCR. Western blot and annexin V-FITC staining were performed to investigate the role of MCM3AP-AS1/SIRT1 in ARPE-19 cell proliferation and apoptosis in vitro.

**Results:**

We observed that MCM3AP-AS1 was downregulated in DR patients 25 comparing to T2D patients without significantly complications. Bioinformatics analysis showed that MCM3AP-AS1 might bind miR-211. However, no significant correlation between these two factors was observed in DR patients. Consistently, overexpression of MCM3AP-AS1 and miR-211 failed to affect the expression of each other in hRPE. Interestingly, MCM3AP-AS1 overexpression upregulated SIRT1, a target of miR-211. Moreover, MCM3AP-AS1 was downregulated in DR patients compared to type 2 diabetic mellitus patients without significant complications. In RPEs, high glucose treatment downregulated MCM3AP-AS1. Cell apoptosis analysis showed that MCM3AP-AS1 and SIRT1 overexpression decreased the apoptotic rate of RPEs, and miR-211 overexpression reduced the effect of MCM3AP-AS1 and SIRT1 overexpression.

**Conclusion:**

MCM3AP-AS1 is downregulated in DR and promotes cell apoptosis by regulating miR-211/SIRT1.

## Background

Diabetes is the most common type of metabolic disorder worldwide [1]. With the people’s lifestyle changing and the aging population growing, the incidence of diabetes is predicted to increase [1, 2], leading to increased economic burden [2]. In 2017, the estimated economic cost of diabetes in the US was $327 billion, including $90 billion of reduced productivity and $237 billion of direct medical costs [3]. Curative therapeutic approaches for diabetes remain lack, and the continuous progression of the disease will cause the development of multiple complications, such as diabetic retinopathy (DR) [4], which affects more than 30% of diabetic patients and may cause blindness in extreme cases even after active treatment [2, 5], leading to heavy economic and psychological burdens on patients and their families.

Studies on the molecular pathogenesis of DR have characterized a considerable number of molecular pathways involved in the development and progression of DR [6]. The identification of molecular players in DR provided novel insights into the development of preventative and treatment approaches [7]. As small noncoding RNAs composed of ~ 19–25 nucleotides, miRNAs were reported to be involved in glucose metabolism and diabetic development by regulating endothelial function and inflammatory processes. A previous study indicated that both miR-21-5p and miR-126-3p were found to contribute to the inflammation and endothelial dysfunction in type 2 diabetic mellitus (T2DM) patients, predicting their future use in estimating the risk of occurrence of T2DM complications. In addition, several miRNAs, including miR-146a, miR-15a, and miR-375, are used as diagnostic or prognostic markers before the onset of T2DM and pre-DM, which may significantly improve the diagnostic accuracy of T2DM. Wang et al. recently characterized a novel miR-211/SIRT1 pathway that is involved in regulating cell apoptosis in DR [8]. It is known that the functions of miRNAs can be regulated by certain long (> 200nt) noncoding RNAs (lncRNAs) [9]. Some studies reported that MCM3AP-AS1 is involved in the development and progression of various human cancers and plays important roles in cell proliferation, invasion, migration, and chemosensitivity [10]. Previous studies indicated that the interaction between MCM3AP-AS1 and miR-211 plays a prominent role in the regulation of glioblastoma angiogenesis. Vascular complications remain the principal cause of morbidity and mortality during diabetes, including diabetic nephropathy, diabetic cardiomyopathy, and DR. By conducting bioinformatic analysis, we confirmed that miR-211 was a potential target of MCM3AP-AS1. Considering the crucial role of miR-211 in DR and its interaction with lncRNA MCM3AP-AS1 in stimulating angiogenesis, we hypothesized that they might involve in the development of DR. Therefore, the current study was carried out to investigate the expression levels and biological functions of MCM3AP-AS1 and miR-211, and their interaction between in DR. To our best knowledge, this is the first report on the expression and biological role of MCM3AP-AS1 in DR.

## Materials and methods

### Research subjects

All participants were admitted to the Sixth Affiliated Hospital, Sun Yat-sen University from March 2016 to March 2019. The study passed the review of the Ethics Committee of the Sixth Affiliated Hospital. The research subjects included 80 proliferative DR patients (45 males and 35 females, 41 to 69 years, 55.1 ± 6.4 years), 80 T2DM patients without complications (45 males and 35 females, 40 to 69 years, 55.5 ± 6.1 years), and 80 healthy controls (45 males and 35 females, 42 to 68 years, 55.2 ± 6.2 years). The patients with diabetes mellitus were diagnosed if they had 1) a fasting plasma glucose (FPG) level of 126 mg/dL (7.0 mmol/L) or higher, 2) a 2-h plasma glucose level of 200 mg/dL (11.1 mmol/L) or higher during a 75-g oral glucose tolerance test (OGTT), or 3) an hemoglobin A1c level of equal or greater than 6.5%. All patients were examined for visual function and ocular anterior and posterior segments. The patients with metabolic diseases (e.g., dyslipidemia), malignancies, pregnancy, use of medications (e.g., anti-inflammatory drugs and corticoids) and alcohol, or severe chronic diseases such as infections, cardio-cerebrovascular events, and hepatic and renal insufficiency, were excluded from this study. The healthy controls were selected to match the distributions of age and gender of patient groups. All patients were informed about the experimental principle. All patients signed informed consent. Clinical data of the three groups of participants were presented in Table 1.

**Table 1:** Clinical data of the three groups of participants

	DR	T2DM	Control
Cases	80	80	80
Gender			
Male (n)	35	35	35
Female (n)	45	45	45
Average age (years)	25.4 ± 1.3	25.1 ± 1.5	23.7 ± 1.1
Duration of diabetes (years)	7.4 ± 2.4	6.9 ± 1.9	NA
DBP	85.78 ± 6.75	86.18 ± 7.75	83.78 ± 6.55
SBP	136.15 ± 13.27	138.15 ± 11.58	132.15 ± 10.27
FBG	8.95 ± 1.65*	8.93 ± 1.34*	4.73 ± 0.83
HBA1c (%)	7.95 ± 1.23*	7.51 ± 1.27*	5.17 ± 0.83

DR, Diabetic Retinopathy; T2DM, Type 2 diabetic mellitus; FBG, fasting blood glucose; HbA1c, glycated hemoglobin; DBP, diastolic blood pressure; SBP, systolic blood pressure; *P < 0.001 vs. control group

### Plasma and ARPE-19 cells

Fasting peripheral blood samples (5 ml) were withdrawn from DR patients, T2DM patients, and controls into EDTA tubes and centrifuged for 20 min at 1200*g* to collect plasma samples. Plasma samples were stored in a liquid nitrogen tank before use. The hemolyzed plasma samples, which could be visually identified as a pink to red color, were excluded.

Human retinal pigment epithelial cell (hRPE) line ARPE-19 (ATCC, USA) was used in this study. Cells were cultured in a mixture composed of 10% FBS and 90% DMEM:F12 medium at 37 °C in an incubator with 5% CO_2_ and 95% humidity.

### Transient cell transfections

MiR-211 mimic and negative control (NC) miRNA were from Sigma-Aldrich (USA). SIRT1 and MCM3AP-AS1 expression vectors (pcDNA3.1 vector as the backbone) were established by Sangon (Shanghai, China). Various concentrations of the vector (0.1, 1, 10, 20, 30, 40, 50, 60 nM) and incubation time (12, 24, 48 h) were selected to determine the optimum conditions in the transient transfections. At last, hRPE cells were harvested at 75–85% confluence, and 3 × 10^6^ cells were transfected with 10 nM vector (empty pcDNA3.1 vector as NC group) or 50 nM miRNA (NC miRNA as NC group). All transfections were mediated by Lipofectamine 2000 reagent (Thermo Fisher Scientific). The interval between transfections and following experiments was 24 h. Control (C) cells were untransfected cells.

### RNA and qPCR

RNAzol reagent (Sigma-Aldrich) was used to extract RNAs from plasma and ARPE-19 cells following the instructions from Sigma-Aldrich except that 85% ethanol was used to precipitate RNAs to harvest miRNAs. To achieve high glucose treatment, ARPE-19 cells were cultured in the culture medium mentioned above with supplementation of 5, 15, 30, and 50 mM D-glucose for 24 h before RNA extractions.

Tetro Reverse Transcriptase system (Bioline) was used to perform all reverse transcriptions (RTs) with ploy (T) as the primer and RNA samples as the template. All qPCR reaction mixtures were prepared using KAPA SYBR FAST qPCR Master Mix (Kapa Biosystems) to measure the expression levels of SIRT1 mRNA and MCM3AP-AS1 with GAPDH as the endogenous control.

Levels of mature miR-211 expression were measured by performing the addition of poly (A), miRNA RT, and qPCR. All steps were completed using All-in-One™ miRNA qRT-PCR Reagent Kit (GeneCopoeia) with U6 as the endogenous control. The qRT-PCR was followed by a melt curve analysis to determine the specificity of the PCR products and the absence of primer dimer formation. The amplification integrity of each candidate gene was confirmed by electrophoresing the PCR products on a 2% agarose gel; the gel was then observed under UV light (L Pix Molecular Imaging; Loccus Biotecnologia, São Paulo, SP, Brazil).

The MIQE guidelines see Bustin et al. 2009 [11].

PCR reaction conditions: 95 ℃ for 300 s; 95 ℃ for 5 s, 55℃ for 10 s, 72 ℃ for 20 s, 40 cycles; 95 ℃ for 10 s, 60 ℃ for 60 s, 95 ℃ for 1 s, the dissolution curve is derived from the change of the fluorescence signal.

In RT-qPCR experiments, in order to correct the experimental errors caused by factors such as RNA quality, reverse transcription efficiency and PCR enzyme amplification efficiency during the experiment, actin gene (actin), glyceraldehyde-3 Housekeeping genes with stable expression, such as phosphate dehydrogenase gene (GAPDH) and 18S rRNA gene, were used as internal reference genes. However, because these genes are much larger than miRNAs, they are not suitable as internal reference genes for miRNART-qPCR detection. It has been reported that U6 snRNA is very conserved, and it is widely used as an internal reference genes for miRNA RT-qPCR in various species [12].

All reactions were repeated 3 times, and data were processed using the 2^−ΔΔCt^ method. The primers used in the qRT-PCR assay were MCM3AP-AS1 forward 5′-TCCC CTCTTGAGCACACTCT-3′ and reverse 5′-TTCTTGGTTCAGCCCCTTGT-3′; miR-211 forward 5′-TGCGCTTCCCTTTGTCATCCTT-3′ and reverse 5′-CTC AAGTGTCGTGGAGTCGGCAA-3′; SIRT1 forward 5′-TAGCCTTGTCAGATAAG GAAGGA-3′ and reverse 5′-ACAGCTTCACAGTCAACTTTGT-3′; GAPDH forward: 5′-CACATCGCTCAGACACCATG-3′ and reverse 5′-TGACGGTGCCATGGAATT TG-3′, and U6 forward 5′-CTCGCTTCGGCAGCACA-3′ and reverse 5′-AACGCTTCACGAATTT GCGT-3′.

### Western blot

ARPE-19 cells were harvested and counted. Cell pellets containing 10^5 ^cells were resuspended in 1 ml RIPA solution (Sigma-Aldrich) to extract proteins. All protein samples were quantified using BCA assay (Sigma-Aldrich). About equal amounts of proteins were denatured at 95 °C for 10 min, separated on 12% SDS-PAGE gel, and transferred onto PVDF membranes. The membranes were blocked in PBS containing 5% non-fat milk and incubated with SIRT1 (ab12193, 1:2000; Abcam) and GAPDH (ab9485, 1:2000, Abcam) rabbit polyclonal primary antibodies for 16 h at 4 °C. After that, membranes were further incubated with IgG-HRP (1:2000, MBS435036, MyBioSource) secondary antibody for 2 h at room temperature. Signals were produced using ECL (Sigma-Aldrich, USA) and processed using Image J v1.46 software.

### Cell apoptosis analysis

ARPE-19 cells were harvested and counted. Cell pellets containing 10^5^ cells were resuspended in 10 ml medium to prepare single-cell suspensions, followed by the addition of 50 mM D-glucose. Cells were cultured under the aforementioned conditions for 48 h. Following 0.25% trypsin digestion, cells were stained with propidium iodide (PI) and annexin V-FITC and subjected to flow cytometry to separate apoptotic cells.

### Statistical analysis

We performed power calculations before the start of the study. If 10% of the patients in the intervention group developed during the first year and we applied a power of 80% and α of 0.05, we would need a minimum of 146 patients. Taking into account the dropouts (10%), we had to include 160 patients. Three biological replicates were included in each experiment. Mean values were calculated and used in all statistical analyses. Linear regression was performed to analyze correlations. ANOVA (one-way) and Tukey tests were used to analyze differences among multiple groups. *P* < 0.05 was statistically significant.

## Results

### MCM3AP-AS1 was downregulated in DR patients and by high D-glucose in ARPE-19 cells

**Fig. 1:**
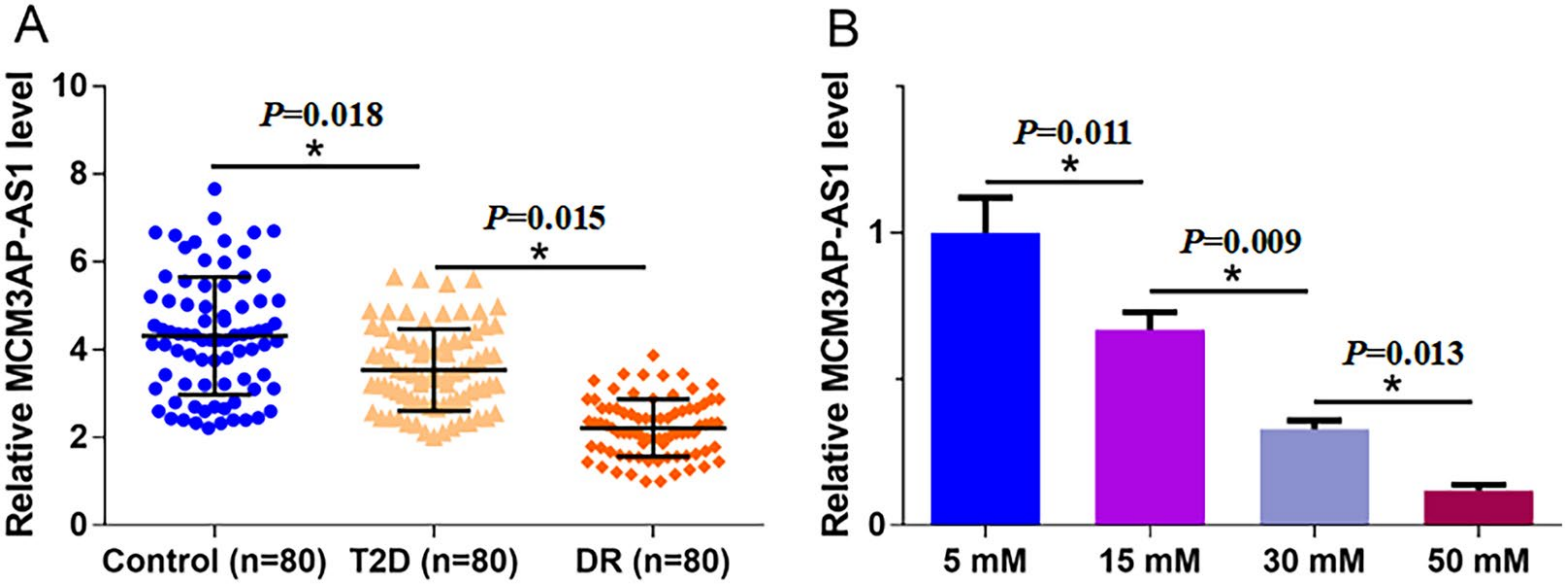
MCM3AP-AS1 was downregulated in DR patients and downregulated by high D-glucose in ARPE-19 cells. Plasma levels of MCM3AP-AS1 in DR patients (n = 80), T2DM patients (n = 80), and Controls (n = 80) were measured by qPCR, and data comparisons were performed using ANOVA (one-way) and Tukey test (**A**). ARPE-19 cells were cultured in a medium containing 5, 15, 30, and 50 nM D-glucose for 24 h, followed by the measurement of MCM3AP-AS1 expression levels using qPCR (**B**). PCR reactions were repeated 3 times, and mean values were presented. *p < 0.05

Plasma MCM3AP-AS1 levels in DR patients (n = 80), T2DM patients (n = 80), and Controls (n = 80) were measured by qPCR and compared using ANOVA (one-way) and Tukey test. Plasma MCM3AP-AS1 level was significantly lower in DR and T2DM groups than in the Control group (Fig. 1A, p < 0.05) and significantly lower in DR patients than in T2DM patients (Fig. 1A, p < 0.05). In addition, MCM3AP-AS1 level in ARPE-19 cells cultured in media containing 5, 15, 30, or 50 mM D-glucose for 24 h was also measured using qPCR. It was observed D-glucose treatment downregulated MCM3AP-AS1 expression in a dose-dependent manner (Fig. 1B, p < 0.05).

### The interaction between MCM3AP-AS1 and miR-211

**Fig. 2:**
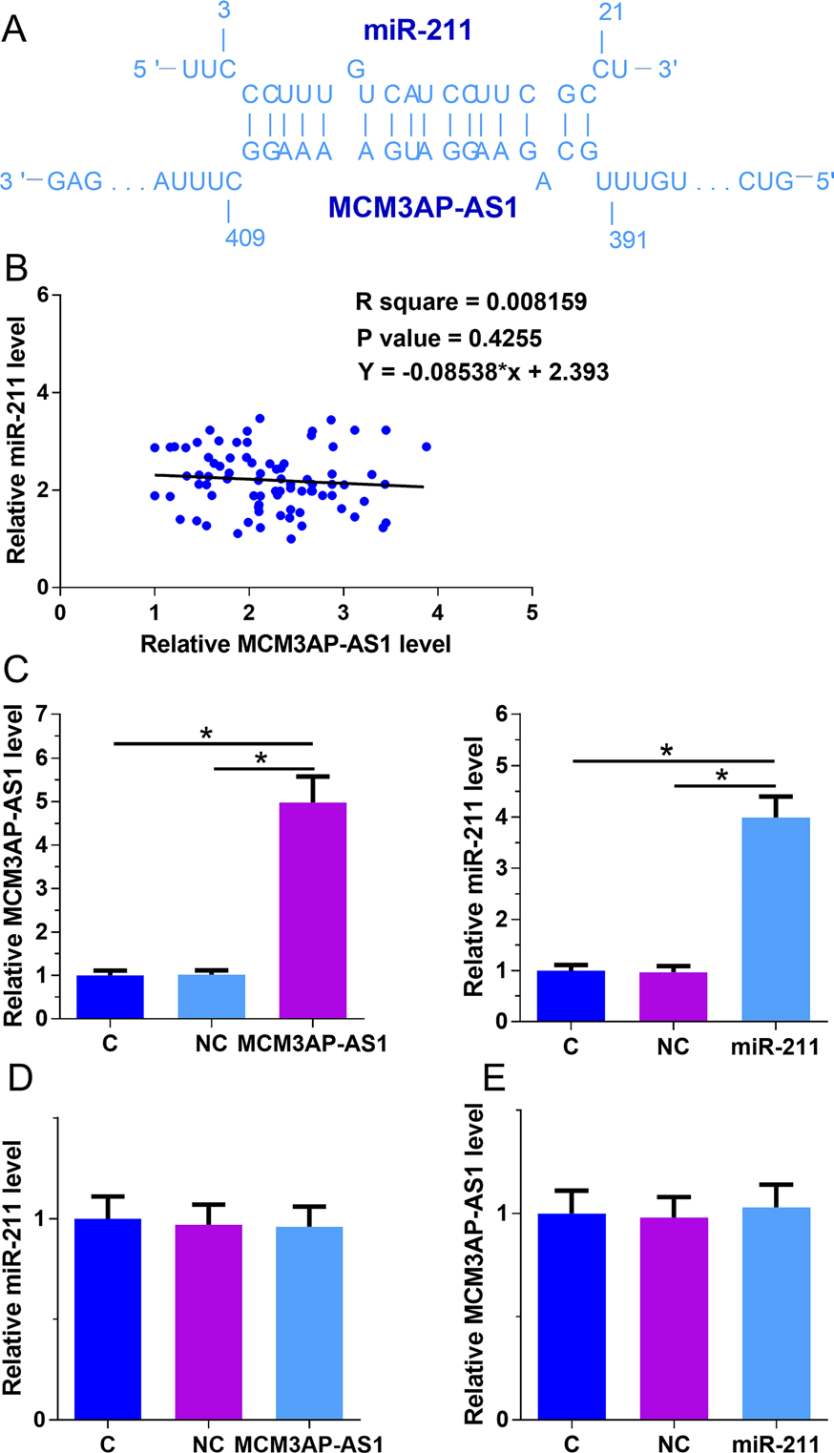
MCM3AP-AS1 may interact with miR-211, but they failed to regulate the expression of each other. The interaction between MCM3AP-AS1 and miR-211 was predicted using IntaRNA (http://rna.informatik.uni-freiburg.de/IntaRNA/Input.jsp). It was observed that miR-211 could form a strong base-pairing with MCM3AP-AS1 (**A**). Levels of miR-211 in plasma of DR patients (n = 80) were measured by qPCR. The correlation between MCM3AP-AS1 and miR-211 was analyzed by linear regression (**B**). ARPE-19 cells were transfected with MCM3AP-AS1 vector and miR-211 mimic. Overexpression of MCM3AP-AS1 and miR-211 was confirmed by qPCR at 24 h post-transfection (**C**). The effects of MCM3AP-AS1 overexpression on miR-211 expression (**D**) and the effects of miR-211 overexpression on MCM3AP-AS1 expression (**E**) were analyzed by qPCR. Experiments were repeated 3 times, and data were expressed as mean values. *, p < 0.05

The interaction between MCM3AP-AS1 and miR-211 was predicted using IntaRNA (http://rna.informatik.uni-freiburg.de/IntaRNA/Input.jsp). It was observed that miR-211 could form strong base-pairing with MCM3AP-AS1 (Fig. 2A). Plasma miR-211 levels in DR patients (n = 80) were measured by qPCR. The correlation between MCM3AP-AS1 and miR-211 was analyzed by linear regression. The results showed that MCM3AP-AS1 and miR-211 were not significantly correlated with each other (Fig. 2B). To further analyze the interactions between MCM3AP-AS1 and miR-211, ARPE-19 cells were transfected with MCM3AP-AS1 vector and miR-211 mimic. Overexpression of MCM3AP-AS1 and miR-211 was confirmed by qPCR at 24 h post-transfection (Fig. 2C, p < 0.05). Comparing to C and NC groups, MCM3AP-AS1 overexpression failed to affect miR-211 expression (Fig. 2D), and miR-211 overexpression failed to affect MCM3AP-AS1 expression (Fig. 2E).

### MCM3AP-AS1 overexpression led to upregulated SIRT1

**Fig. 3:**
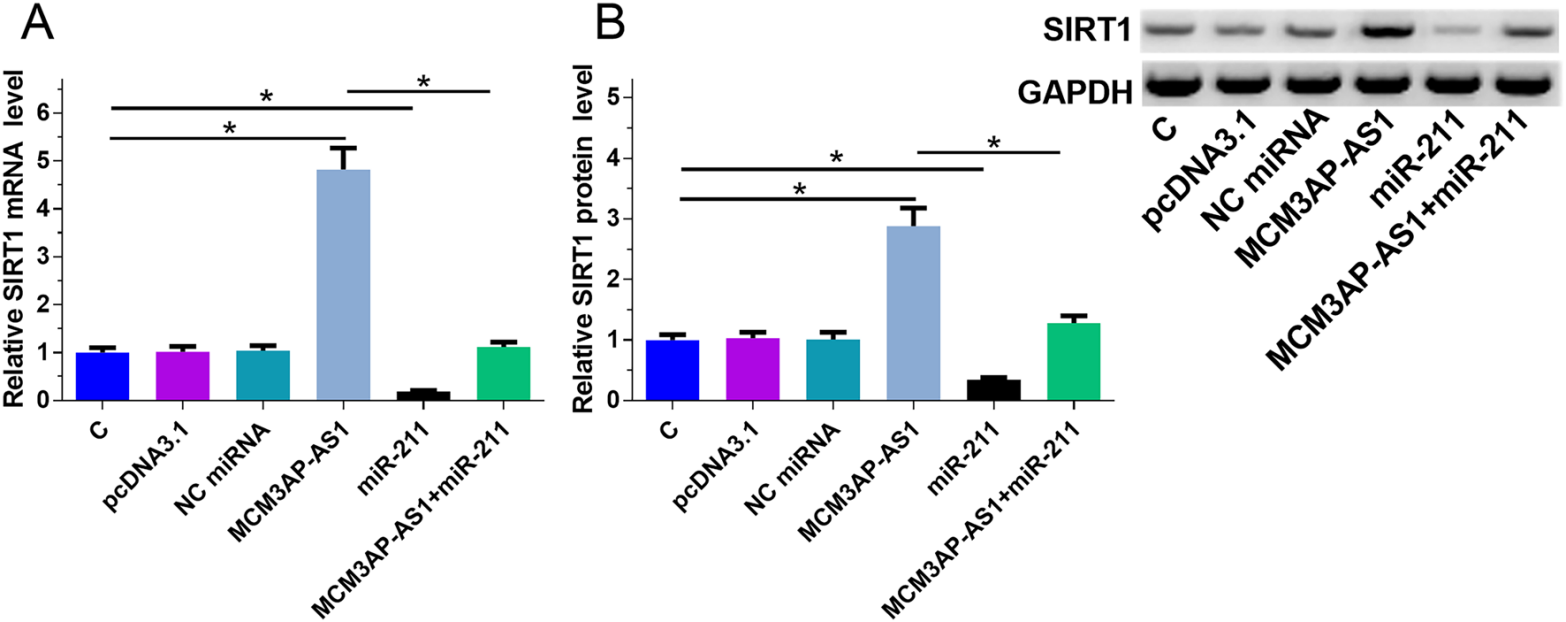
MCM3AP-AS1 overexpression led to upregulated SIRT1. QPCR and Western blot were performed to analyze the effects of MCM3AP-AS1 and miR-211 overexpression on the expression of SIRT1 at both mRNA (**A**) and protein (**B**) levels, respectively. Experiments were repeated 3 times, and data were expressed as mean values. *p < 0.05

QPCR and Western blot were performed to analyze the effects of MCM3AP-AS1 and miR-211 overexpression on SIRT1 expression at both mRNA (Fig. 3A) and protein (Fig. 3B) levels, respectively. Comparing to C and NC (pcDNA3.1 vector and NC miRNA transfection) groups, MCM3AP-AS1 overexpression upregulated SIRT1, a target of miR-211. MiR-211 overexpression played an opposite role and attenuated the effects of MCM3AP-AS1 overexpression (p < 0.05).

### MCM3AP-AS1 overexpression suppressed ARPE-19 cell apoptosis under high glucose through miR-211 and SIRT1

**Fig. 4:**
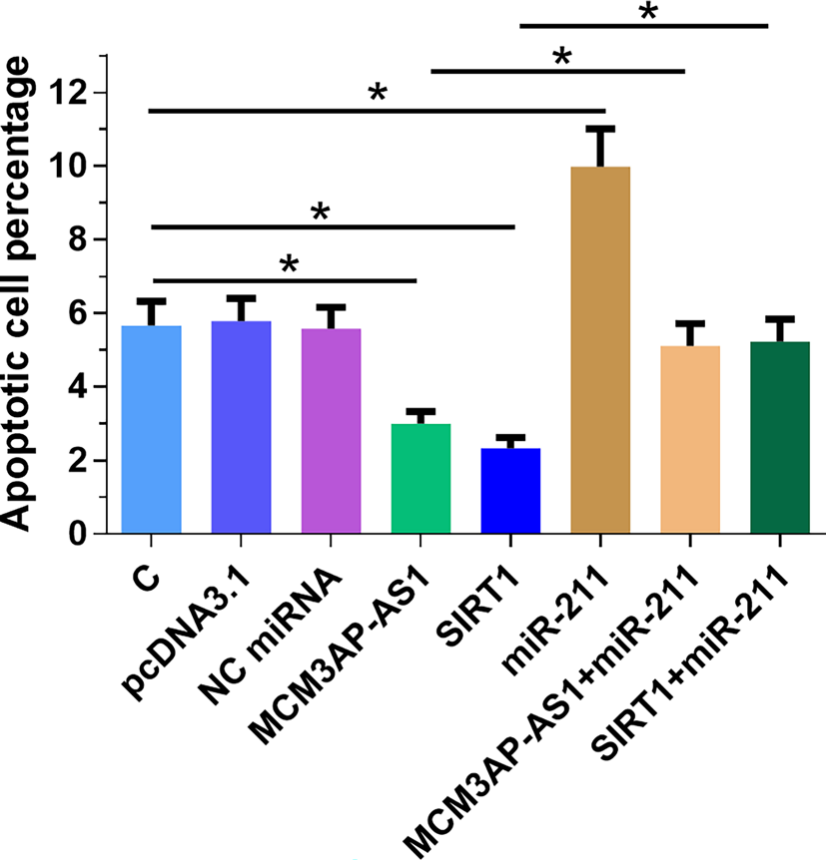
MCM3AP-AS1 overexpression led to suppressed ARPE-19 cell apoptosis under high glucose through miR-211 and SIRT1. Effects of MCM3AP-AS1, miR-211 and SIRT1 overexpression on the apoptosis of ARPE-19 cells under the treatment of 50 mM D-glucose were analyzed by performing cell apoptosis assay. Experiments were repeated 3 times, and data were expressed as mean values. *p < 0.05

The effects of MCM3AP-AS1, miR-211, and SIRT1 overexpression on the apoptosis of ARPE-19 cells treated with 50 mM D-glucose were analyzed by cell apoptosis assay. Compared to C and NC (NC miRNA or empty vector transfection), MCM3AP-AS1 and SIRT1 overexpression decreased the apoptotic rate of ARPE-19 cells. In addition, miR-211 overexpression reduced the effects of MCM3AP-AS1 and SIRT1 overexpression (Fig. 4, p < 0.05).

## Discussion

This study is the first report of the interaction between lnc RNAs MCM3AP-AS1 and miR-211/SIRT1 in DR patients. We found that MCM3AP-AS1 was downregulated in DR and might sponge miR-211 to upregulate SIRT1, which in turn inhibited cell apoptosis under high glucose conditions. Previous studies have characterized a considerable number of differentially expressed lncRNAs in DR [13]. Some of the differentially expressed lncRNAs have been proven to play critical roles in disease initiation and progression [14, 15]. For instance, lncRNA H19 is overexpressed in DR patients, and its overexpression inhibits high glucose-induced endothelial–mesenchymal transition, thereby improving disease condition [14]. Another study reported that lncRNA MEG3 was downregulated in DR, and its overexpression regulated TGF‑β1 and VEGF signaling to suppress cell apoptosis [14]. Diabetes could affect the component neurovascular unit of the retina, leading to neurodegeneration, neuroinflammation, compromise of the vascular blood-retinal barrier, edema, angiogenesis, and eventual fibrosis [16]. A previous study indicated that MCM3AP-AS1 knockdown and miR-211 overexpression could regulate glioblastoma angiogenesis [17]. Our study showed MCM3AP-AS1 downregulation in DR. In addition, MCM3AP-AS1 overexpression suppressed apoptosis of hRPE cells under high glucose treatment. Therefore, MCM3AP-AS1 might protect vascular blood-retinal barrier in DR. Our study also found that the levels of fasting blood glucose and HbA1c were significantly higher in both DR and T2DM patients than in healthy subjects, but not significantly different between DR and T2DM patients. However, MCM3AP-AS1 expression was markedly downregulated in DR patients than in T2DM patients. Interestingly, high glucose treatment downregulated MCM3AP-AS1 expression in hRPE cells. These findings suggest that MCM3AP-AS1 downregulation in DR and T2DM patients is likely induced by a high glucose environment and might be involved in diabetic complications, such as DR.

The main function of miRNAs is to downregulate downstream target genes by direct targeting [18, 19]. In this study, we showed that miR-211 could form a strong base-pairing with MCM3AP-AS1. However, miR-211 overexpression failed to affect MCM3AP-AS1 expression significantly. Therefore, MCM3AP-AS1 is unlikely a target of miR-211. Besides serving as the target of miRNAs, lncRNAs can also mimic miRNAs’ target to attenuate their effects on downstream genes [20, 21]. SIRT1 is a direct downstream target of miR-211.

There are still some limitations of this study. (1) The experimental conditions of this study need to be further optimized; (2) The sample size in this study is single, and it is expected that multi-center expansion of the sample size can be carried out in future studies; (3) The regulation of MCM3AP-AS1 and miR-211/SIRT1 was also reported in other diseases such as glioblastoma [17], gastric cancer [22], prostate cancer [23]; (4) Although, it has been reported that U6 snRNA is very conserved, however, the U6 functions effectively only for RNA polymerase III transcription. Thus our findings need to be explored in other disease models.

## Conclusion

In this study, we observed the upregulation of SIRT1 after MCM3AP-AS1 overexpression. Therefore, MCM3AP-AS1 might sponge miR-211 to upregulate SIRT1, thereby inhibiting the apoptosis of hRPEs under a high glucose environment.

## Data Availability

The data sets generated during the study are available from the corresponding author on reasonable request.
